# Diabetes mellitus is associated with an increased risk of postoperative neurocognitive disorders: a systematic review

**DOI:** 10.3389/fmed.2026.1726908

**Published:** 2026-02-13

**Authors:** Hong Li, Jing Xue, Zhiyong Gao, Li Xian, Jinlong Yuan, Jing He

**Affiliations:** 1 Department of Anesthesiology, No. 363 Hospital, Chengdu, Sichuan, China; 2 Department of Nursing, No. 363 Hospital, Chengdu, Sichuan, China

**Keywords:** bibliometric analysis, diabetes mellitus, diabetic retinopathy, metabolic disorder, neurocognitive disorder

## Abstract

**Background:**

Diabetes mellitus (DM) is increasingly recognized as an independent risk factor for perioperative neurocognitive disorders (PND). The rising global incidence of DM, projected to affect over 783 million people by 2045, necessitates a deeper understanding of its implications for surgical outcomes.

**Methods:**

This review conducted a bibliometric analysis of 182 studies indexed in the Web of Science, focusing on the relationship between DM and PND. A systematic search was performed across multiple electronic databases, including PubMed and The Cochrane Library, to identify relevant articles published up to November 2024.

**Results:**

The analysis revealed that DM significantly increases the risk of PND, particularly POD, with studies indicating a 1.84-fold increased risk of POCD in diabetic patients. Key mechanisms identified include vascular brain injury, impaired glucose metabolism, inflammation, and oxidative stress. Notably, countries such as China, the USA, and Germany are leading research efforts in this area, highlighting a global interest in understanding these associations.

**Conclusion:**

Further research is needed to elucidate causal mechanisms between DM and PND. Enhanced understanding of these mechanisms may inform targeted interventions to mitigate the risk of cognitive decline in diabetic patients undergoing surgery, ultimately improving patient outcomes and reducing healthcare burdens.

## Introduction

Diabetes mellitus (DM), a chronic metabolic disorder, has emerged as a significant challenge to global public health. According to recent data, the global incidence of diabetes was approximately 22.9 million cases in 2017, a figure that is projected to escalate to 26.6 million by the year 2025 (1). In 2021, the prevalence of diabetes among individuals aged 20–79 years was estimated to be 10.5%, affecting around 536.6 million people worldwide. This trend is expected to continue, with the prevalence rate predicted to climb to 12.2% by 2045, impacting approximately 783.2 million individuals (1). These statistics indicate a growing concern as both the incidence and mortality rates associated with diabetes are on the rise globally, with a particularly sharp increase observed in developing nations (1). This surge is particularly notable among patients with DM who are undergoing various surgical procedures. Perioperative neurocognitive disorders (PND), a common complication associated with surgical anesthesia, encompasses postoperative delirium (POD) and postoperative cognitive dysfunction (POCD) (2). The high incidence of PND has a profound impact on postoperative recovery, quality of life, and imposes a substantial economic burden on both society and families, making it a critical public health issue in recent years (2, 3).

The relationship between DM and perioperative neurocognitive disorders (PND) is garnering increasing attention in research circles (4, 5). Studies have demonstrated that diabetic patients are at a higher risk of developing PND compared to their non-diabetic counterparts (6). Intriguingly, an analysis of the Medical Information Mart for Intensive Care (MIMIC)-IV database suggests that focusing on various blood glucose metrics, such as mean blood glucose levels, mean absolute glucose, mean amplitude of glycemic excursions, and glycemic lability index, may provide a more comprehensive assessment of the potential risk for postoperative delirium (POD) in diabetic patients undergoing surgery, rather than relying solely on blood glucose levels at ICU admission (7).

Furthermore, research has begun to shed light on the potential mechanisms linking DM and PND, suggesting that this association could involve a complex interplay of factors, including vascular brain injury, impaired brain glucose metabolism, inflammation, oxidative stress, and elevated levels of tau biomarkers (7–11). While these studies have made strides in understanding the correlation between DM and PND, a comprehensive bibliometric analysis of the most current trends remains limited. Using the PICO framework, we included adult surgical patients with pre-existing diabetes mellitus (P), does exposure to any peri-operative glycemic or anti-diabetic intervention (I), compared with non-diabetic surgical patients or diabetic patients without such intervention (C), influence the incidence of post-operative delirium or cognitive dysfunction (O)? Existing narrative reviews have summarized selected clinical studies, but none have quantitatively mapped the global research landscape, trend-sets, or mechanistic clusters. By coupling bibliometric analysis of 182 studies with a focused systematic appraisal of 23 high-data publications, the present review is the first to integrate geographical output, hotspot evolution and risk-quantification, thereby guiding future mechanistic and interventional work rather than duplicating earlier summaries.

## Materials and methods

### Searching strategy and data collection

This study adhered to the stringent guidelines outlined in the Preferred Reporting Items for Systematic Reviews and Meta-Analyses (PRISMA) statement (12). Our comprehensive search spanned across several reputable electronic databases, including PubMed, The Cochrane Library, and the Web of Science (WOS) citation index, from their inception up to August 2024. Our aim was to identify articles that align with our predefined inclusion criteria. Titles and abstracts were independently screened by two reviewers; disagreements were resolved by a third reviewer. Data extraction was performed independently by two reviewers using a pre-piloted form.

The search strategy incorporated a set of carefully curated terms to ensure a thorough and relevant selection of literature. These terms included “postoperative neurocognitive disorders,” “perioperative neurocognitive disorders,” “postoperative delirium,” “postoperative cognitive decline,” and “postoperative cognitive dysfunction,” which were combined using Boolean logic with the terms “preoperative diabetes,” “diabetes,” and “diabetes mellitus.” This approach allowed us to filter the literature effectively and identify studies that explore the intersection of preoperative diabetes and postoperative cognitive outcomes.

It is important to note that our search was not confined by language barriers; we sought to include all relevant studies regardless of the language in which they were published. This inclusive approach was designed to ensure a broad and diverse range of perspectives and findings. The detailed search strategy, including the specific terms and their combinations, is provided in the Supplementary Digital Content, offering transparency and reproducibility to our methodology.

### Study selection criteria

The literature search and screening process for this study was conducted meticulously and independently by two researchers to ensure rigor and reliability. Any discrepancies that emerged during this process were resolved through consensus, which is a standard practice to maintain the integrity of the study’s methodology.

The inclusion criteria for the bibliometric analysis in this study were stringent and well-defined. Firstly, the study focused on English-language full articles that had been published, which included a variety of research designs: randomized controlled trials (RCTs), observational studies (encompassing both prospective and retrospective cohorts), case reports, reviews, meta-analyses, and protocol studies. The primary criterion for inclusion was that these publications must explore the role of diabetes mellitus (DM) in perioperative neurocognitive disorders (PND). This broad inclusion of study types allows for a comprehensive analysis of the existing body of literature on this topic. As a result, 182 studies indexed in the Web of Science (WOS) were identified and included for an analysis of publication trends, indicating the extensive scope of the research conducted in this area.

Secondly, to gain a deeper understanding of clinical research and the mechanisms by which DM influences PND, the study homed in on articles that provided detailed data. This included RCTs and observational studies, which were systematically analyzed to examine subset research within the broader field. By focusing on these detailed studies, the researchers aimed to uncover more nuanced insights into the relationship between DM and PND, contributing to a more profound understanding of this complex issue.

## Results

**FIGURE 1:**
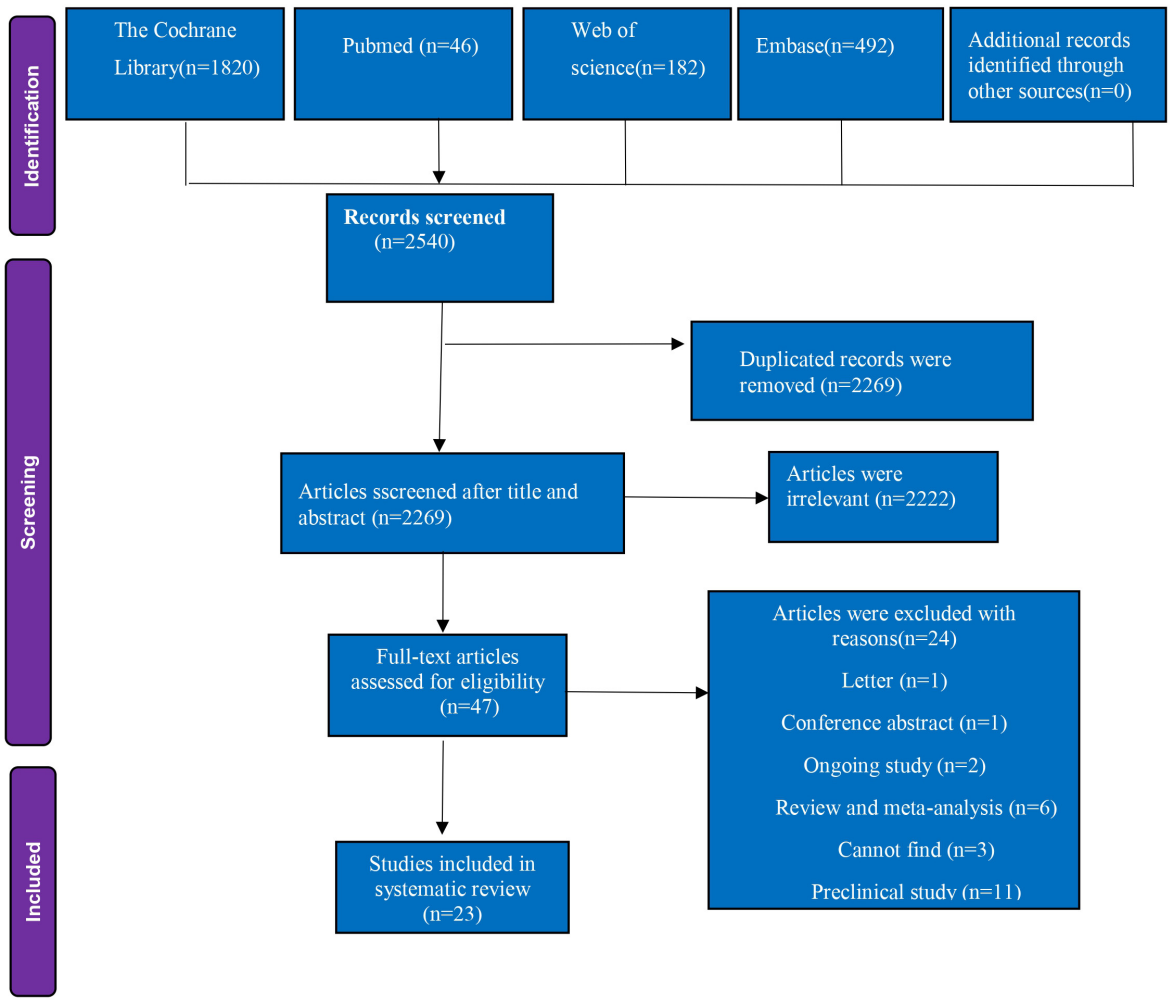
Flow diagram of included and excluded studies.

The PRISMA (Preferred Reporting Items for Systematic Reviews and Meta-Analyses) flow diagram, as depicted in Figure 1, provides a visual representation of the meticulous process we undertook to select studies for our systematic review. Commencing an extensive literature search, we scoured four reputable electronic databases, yielding an initial pool of 2,540 citations. This collection was then subjected to a rigorous screening process, which involved the application of predefined inclusion and exclusion criteria to ensure the relevance and quality of the studies. Following this meticulous curation, we narrowed down the vast array of citations to a curated list of 24 studies that met all our specified criteria. These selected studies underwent a systematic analysis, the details of which are comprehensively outlined in Table 1, offering a transparent account of our study selection process and the evidence base for our review.

**TABLE 1:** The demographic information of included studies.

References	Region/country	Study design	No. patients	Age (years)	Surgery	PND/POD/POCD prevalence or incidence (%)	Cognitive assessment tools	Findings
Kadoi et al. (20)	Japan	Clinical study	90	65 ± 9	CABG	Cognitive dysfunction was 50% at 7 days and 23% at 6 months postoperatively	1. MMSE; 2. Rey auditory verbal learning test; 3. Trail-making test (part A and B); 4. Digit span forward; 5. Grooved pegboard	The presence of depression preoperatively is associated with short-and long-term postoperative cognitive dysfunction in patients with DM.
Kadoi et al. (21)	Japan	Clinical study	120	67 ± 4	CABG	Cognitive dysfunction in the impaired CO_2_ group was 57%	1. MMSE; 2. Rey auditory verbal learning test; 3. Trail-making test (part A and B); 4. Digit span forward; 5. Grooved pegboard	Impaired cerebrovascular CO_2_ reactivity was associated with postoperative short-term cognitive dysfunction in DM patients.
Kadoi et al. (5)	Japan	Clinical study	180	64 ± 11	CABG	–	1. MMSE; 2. Rey auditory verbal learning test; 3. Trail-making test (part A and B); 4. Digit span forward; 5. Grooved pegboard	Insulin therapy, diabetic retinopathy, and hemoglobin A1c were factors in cognitive impairment at 7 days and 6 months after CABG in patients with DM.
Kadoi et al. (22)	Japan	Prospective, observational study	99	62 ± 6	CABG	51% at 7 days and 26% at 6 months postoperatively	1. MMSE; 2. Rey auditory verbal learning test; 3. Trail-making test (part A and B); 4. Digit span forward; 5. Grooved pegboard	Amelioration of SjvO2 values was not associated with short- or long-term POCD in DM patients with impaired CO_2_ reactivity.
Kotfis et al. (17)	USA	Observational cohort study	1,010	70.09 ± 8.43	Cardiac surgery	502 (15.8%) patients were diagnosed with POD.	DSM-5	Elevated preoperative HbA1c level is a risk factor for postcardiac surgery delirium regardless of the diagnosis of DM.
Lachmann et al. (23)	Germany	Observational study	1,034	1. 61.0 ± 9.1 2. 64.7 ± 11.6 3. 69.5 ± 6.3	Cardiac and non-cardiac surgery	DM was associated with a 1.84-fold increased risk of POCD.	Several age-sensitive neuropsychological tests	Consideration of DM status may be helpful for risk assessment of surgical patients.
Lin et al. (24)	China	Observational study	257	1. 53.3 ± 10.5 2. 51.1 ± 12.8	Acute aortic dissection	103 (38.9%) patients with delirium.	CAM-ICU	Glucose variability is associated with the risk of delirium and high glycemic variability increases the risk of POD.
Liu et al. (25)	China	Secondary analysis	309	1. 81 (75–85) 2. 79 (73–83)	Hip fracture surgery	52 (16.83%) patients experienced POD.	CAM	Older Type 2 DM patients develop POD after hip fracture surgery than patients without Type 2 DM.
Nötzold et al. (26)	Germany	Open cohort study	34	62.44 ± 7.52	CABG	–	Several neurocognitive tests	The cognitive outcome in the early postoperative period is worse in DM patients compared to non-diabetics.
Ntalouka et al. (27)	Greece	Prospective cohort study	144	1. 66.4 ± 7.5 2. 66.6 ± 7.4	Elective non-cardiac surgery	PND (*n* = 96) and higher POD up to 4 days (*n* = 204).	1. CAM 2. IQCODE-16	Patients with type 2 DM appear to be at a higher risk of PND up to 9 months after surgery.
Oh et al. (28)	Republic of Korea	Cohort study	61,805	1. 64.7 (± 14.6) 2. 57.5 (± 14.6)	Non-cardiac surgery	Development of delirium was 1.35 (95% CI, 1.18–1.56) in hyperglycemia	CAM	Hyperglycemia was consistently associated with POD regardless of the presence of DM.
Panidapu et al. (29)	India	Prospective, observational study	100	1. 61.6 ± 6.5 2. 62.2 ± 7.7	Cardiac surgery	1. 3 (6%) in Dexmedetomidine group 2. 10 (20%) in control group	–	Dexmedetomidine infusion during the intraoperative period was very effective for perioperative glycemic control and reduction of incidence of POD in DM patients.
Paredes et al. (30)	USA	Retrospective cohort analysis	10,662	1. 63.0 ± 11.5 2. 63.4 ± 12.4	Non-cardiac surgery	312 (6.7%) cases among metformin users had POD and 455 (7.6%) cases among non-metformin users had POD.	CAM-ICU	Chronic metformin use was associated with slightly and non-significantly less delirium in DM patients.
Saager et al. (31)	USA	RCT	198	1. 65 ± 15 2. 66 ± 12	Cardiac surgery	The incidence of delirium was 28% (26 of 93) and 14% (15 of 105) in the hyperinsulinemic-normoglycemic clamp group and the standard therapy group, respectively.	CAM	Tight intraoperative glucose control contributed to a statistically and clinically significant increase in POD.
Sacli and Kara (32)	Turkey	Prospective observational study	54	1. 62 ± 7.7 2. 59.8 ± 9.1	CABG	Mild cognitive dysfunction was observed in 13 (54.2%) patients in Group 1 and in 5 (19.2%) patients in Group 2.	MoCA	The use of advanced neuromonitoring methods can significantly prevent this decrease in cognitive functions in DM.
Shang et al. (33)	China	Prospective matched cohort study	138	1. 69 (64–73) 2. 68 (65–72)	Orthopedic surgery	The incidence of POD was 16.7% (22/132) in patients with preoperative DM patients.	1. 3D-CAM 2. CAM-Severity	Preoperative DM is associated with an increased risk of POD in older patients, and that low intraoperative alpha power partially mediates such association.
Song et al. (6)	China	Retrospective cohort study	236	1. 71.3 ± 5.0 2. 72.4 ± 4.9	Non-cardiac elective surgery	The incidence of PND was significantly higher in DM patients within 30 days 59.2%	1. CAM-ICU 2. MoCA	The incidence of PND is higher in DM than in non-DM patients in China, and preoperative MoCA is an independent risk factor for PND in DM. Meanwhile, the changes in GFAP and p-Tau in DM patients who experienced PND were significantly higher than in non-DM.
Sun et al. (34)	China	Retrospective cohort study	4,566	70.0 (67.0–74.0)	Non-neurosurgery and non-cardiac surgery	The overall incidence of POD in the study participants was 3.6%. The group with TyG > 8.678 exhibited a higher incidence of POD.	DSM-4	The TyG index shows promise as a novel biomarker for predicting the occurrence of POD in elderly surgical patients with DM.
van Zuylen et al. (35)	Netherlands	Prospective cohort study	102	72.0 (68–74)	Elective surgery	DM patients with DNR (*n* = 11, 50%)	TICS-M	Older adult patients with DM undergoing surgery have an increased risk of DNR compared to older adult non-DM patients, but no increased risk of POCD.
Wang and Mei (36)	China	Retrospective cohort study	1,951	68.0 (61.0–74.0)	CABG	180 (9.2%) patients had POD	CAM-ICU	Focusing on levels of MBG, MAG, GLI, and MBG trajectory may be more beneficial to assess the potential risk of POD than the blood glucose level upon ICU admission in patients with DM.
Windmann et al. (37)	Germany	Prospective, observational cohort	87	74 (69–77)	Elective surgery	POD occurred in 41 (47.1%), POCD in five (15.2%) patients.	1. DSM-5 2. Nu-DESC 3. CAM 4. CAM-ICU	Intraoperative hyperglycemia was independently associated with POD but not POCD.
Yang et al. (38)	China	Clinical study	52	1.72.5 ± 3.8	Elective surgery	The incidence of POCD in patients with POD was 57.2% higher than that in patients without POD (*P* = 0.019)	1. CAM-ICU 2. MoCA	There is a significant correlation between the low alpha wave power of EEG during operation and the occurrence of POD in elderly patients with DM.
Zhang et al. (39)	China	Retrospective longitudinal study	855	66.0 ± 12.2	Cardiac surgery	Of the 855 patients included in the study, 271 (31.7%) had new onset neurocognitive disorders at follow-up.	–	Perioperative hyperglycemia was associated with new onset of PND.

CABG, coronary artery bypass graft; MMSE, mini-mental state examination; DSM-5, Diagnostic Statistical Manual of Mental Disorders, fifth edition criteria; CAM-ICU, Confusion Assessment Method for the Intensive Care Unit; CAM, Confusion Assessment Method; IQCODE-16, 16-item Informant Questionnaire on Cognitive Decline; RCT, randomized controlled trial; MoCA, Montreal Cognitive Assessment; 3D-CAM, 3-min Diagnostic Confusion Assessment Method; TICS-M, The Modified Telephone Interview for Cognitive Status Questionnaire; DNR, delayed neurocognitive recovery; MBG, mean blood glucose; MAG, mean absolute glucose; GLI, glycemic lability index; ICU, intensive care unit; TyG, triglyceride-glucose.

### Bibliometric analysis

**FIGURE 2:**
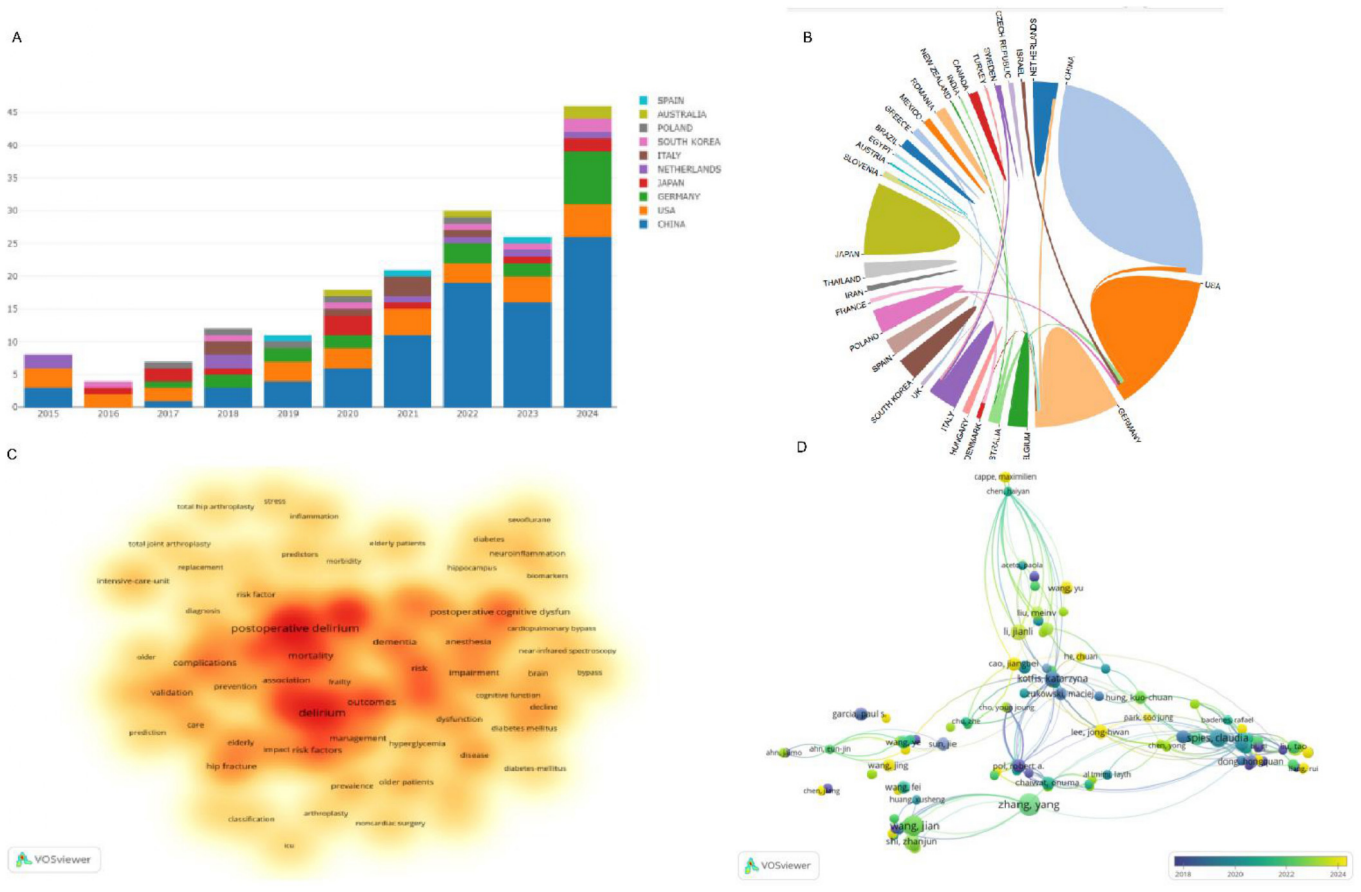
**(A)** Annual publication trends of the top 10 countries in DM on PND research; **(B)** The cooperation network between countries; **(C)** Visualization of frequent keywords use in DM on PND research; **(D)** Presents an overlay visualization network map of authors engaged in DM research on PND. DM, diabetes mellitus; PND, perioperative neurocognitive disorders.

The bibliometric analysis, as presented in the figures, offers a detailed examination of the global research landscape in the field of diabetes mellitus (DM) in postoperative delirium (PND). Figure 2A illustrates the leadership position of China in this research domain, with the United States and Germany closely following in terms of contributions. This visual representation underscores the prominence of these countries in shaping the discourse on DM in PND.

Figure 2B provides a snapshot of the collaborative ecosystem among nations, showcasing an international commitment to addressing this critical topic. The keywords highlighted in Figure 2C, such as “mortality,” “complications,” “frailty,” “dementia,” and “elderly,” are pivotal terminologies that resonate with the core themes of PND research. These terms reflect the multifaceted nature of the condition, and the complexity of the research involved.

Figure 2D presents an author co-citation network map, which reveals the interconnectedness and collaborative efforts among scholars dedicated to DM research in the context of PND. This network map is a testament to the collaborative spirit that drives scientific progress in this area.

The synthesis of data from Figures 2A–D paints a holistic picture of the evolution and current state of research dynamics in DM and PND. Table 2 identifies the top 10 institutions that have made significant contributions to this field, with four of them being Chinese: Capital Medical University (total publications, TC, *n* = 19), Nanjing Medical University (TC, *n* = 10), Southern Medical University (TC, *n* = 9), and Southeast University (TC, *n* = 7). The other institutions are spread across Poland, Thailand, Japan, Belgium, and Germany, indicating a global distribution of research excellence in this domain.

**TABLE 2:** The most influential institutions.

Institution	Total publications	Total citations	Average citations	Region/country
Capital Medical University	19	2	0.11	China
Nanjing Medical University	10	6	0.6	China
Vanderbilt University	9	9	1.0	
Southern Medical University	9	5	0.56	China
Pomeranian Medical University	8	45	5.63	Poland
Mahidol University	8	9	1.13	Thailand
International University of Health and Welfare	8	0	0	Japan
Southeast University	7	21	3.0	China
University of Leuven	7	0	0	Belgium
Charite-University Medicine of Berlin	6	37	6.17	Germany

Table 3 highlights the most influential journals in PND research on DM management, with a notable presence of English journals such as BMC Anesthesiology (TC, *n* = 64), Journal of Cardiothoracic Surgery (TC, *n* = 4), CNS Neuroscience & Therapeutics (TC, *n* = 4), British Journal of Anaesthesia (TC, *n* = 4), and Journal of Orthopaedic Surgery and Research (TC, *n* = 4). The United States also features prominently with journals like Journal of Clinical Medicine (TC, *n* = 6), Journal of Vascular Surgery (TC, *n* = 4), Anesthesia and Analgesia (TC, *n* = 4), and Medicine (TC, *n* = 4). Additionally, there is a journal from New Zealand, Clinical Interventions in Aging (TC, *n* = 5), which adds to the international flavor of the research landscape.

**TABLE 3:** The most influential journals.

Journal	Total publications	Total citations	Average citations	Region/country	Impact factor (2024)
BMC Anesthesiology	6	6	1	England	2.3
Journal of Clinical Medicine	6	0	0	United States	3
Clinical Interventions in Aging	5	7	1.4	New Zealand	3.5
Journal of Vascular Surgery	4	14	3.5	United States	3.9
Journal of Cardiothoracic Surgery	4	8	2	England	1.5
Anesthesia and Analgesia	4	4	1	United States	4.6
Medicine	4	2	0.5	United States	1.3
CNS Neuroscience & Therapeutics	4	0	0	England	4.8
British Journal of Anaesthesia	4	0	0	England	9.1
Journal of Orthopaedic Surgery and Research	3	12	4	England	2.8

Table 4 lists the most globally cited references on DM management in PND, comprising six clinical studies, three systematic reviews and meta-analyses, and one preclinical study. These studies, including works by Wang et al. (13), Chaiwat et al. (14), Feinkohl et al. (4), Wang et al. (15), Zhu et al. (16), and Kotfis et al. (17), underscore the significant role of DM as a risk factor for postoperative delirium (POD) or PND during the perioperative period. Hermanides et al. (18) further suggests a link between DM and acute perioperative hyperglycemia, which may increase the risk for POD or postoperative cognitive dysfunction (POCD). Zhang et al. (19) contributes to the understanding of the pathophysiological relationship between DM and POCD by revealing that high glucose levels, a hallmark of DM, can exacerbate lipopolysaccharides-induced microglial activation and inflammatory cytokine production through the TLR4/JAK2/STAT3 pathway, offering new insights into the mechanisms underlying the association between DM and cognitive complications in the perioperative setting.

**TABLE 4:** The top 10 global cited reference.

First author and year	Reference	DOI	Article type	Journal	Total citation
Wang, 2018	Incidence and risk factors of postoperative delirium in the elderly patients with hip fracture	10.1186/s13018-018-0897-8	Clinical study	Journal of Orthopaedic Surgery and Research	67
Chaiwat, 2019	Postoperative delirium in critically ill surgical patients: incidence, risk factors, and predictive scores	10.1186/s12871-019-0694-x	Clinical study	BMC Anesthesiology	67
Feinkohl, 2017	Diabetes is associated with risk of postoperative cognitive dysfunction: a meta-analysis	10.1002/dmrr.2884	Meta-analysis	Diabetes-Metabolism Research and Reviews	63
Zhang, 2015	Enhancement of LPS-induced microglial inflammation response via TLR4 under high glucose conditions	10.1159/000373972	Preclinical study	Cellular Physiology and Biochemistry	61
Hornor, 2020	Enhancing the American College of Surgeons NSQIP surgical risk calculator to predict geriatric outcomes	10.1016/j.jamcollsurg.2019.09.017	Clinical study	Journal of the American College of Surgeons	57
Visser, 2015	Predicting postoperative delirium after vascular surgical procedures	10.1016/j.jvs.2015.01.041	Clinical study	Journal of Vascular Surgery	55
Wang, 2015	Risk factors contributing to postoperative delirium in geriatric patients post orthopedic surgery	10.1111/appy.12193	Clinical study	Asia-Pacific Psychiatry	48
Hermanides, 2018	Perioperative hyperglycemia and neurocognitive outcome after surgery: a systematic review	10.23736/S0375-9393.18.12400-X	Review	Minerva Anestesiologica	40
Zhu, 2020	Risk factors for postoperative delirium after spinal surgery: a systematic review and meta-analysis	10.1007/s40520-019-01319-y	Meta-analysis	Aging Clinical and Experimental Research	39
Kotfis, 2019	Diabetes and elevated preoperative HbA1c level as risk factors for postoperative delirium after cardiac surgery: an observational cohort study	10.2147/NDT.S196973	Clinical study	Neuropsychiatric Disease and Treatment	38

### Systematic analysis

The comprehensive analysis presented in Table 1 (5, 6, 17, 20–39) encompasses 23 studies that include a substantial cohort of 84,038 patients, spanning a period from 2005 to 2024. These patients, aged between 53 and 83 years and diagnosed with diabetes mellitus (DM), underwent a variety of surgical interventions. The geographical distribution of these studies is diverse, with four originating from Japan (5, 20–22), three from the United States (17, 30, 31), three from Germany (23, 26, 37), and eight from China (6, 24, 25, 33, 34, 36, 38, 39). The remaining five studies hail from Greece (27), the Republic of Korea (28), India (29), Turkey (32), and the Netherlands (35), respectively.

Kadoi et al. (20) conducted a series of studies investigating the impact of various risk factors on cognitive function in DM patients undergoing coronary artery bypass graft surgery (CABG). Their research indicated that preoperative depression is significantly associated with both short-term (50% at 7 days) and long-term (23% at 6 months) postoperative cognitive dysfunction (POCD) in these patients (20). In another finding, the incidence of POCD in patients with impaired cerebrovascular CO_2_ reactivity was notably high at 57% (21). However, an improvement in jugular venous oxygen saturation (SjvO2) values was not found to be associated with short- or long-term POCD in DM patients with impaired CO_2_ reactivity (22). Furthermore, insulin therapy, diabetic retinopathy, and hemoglobin A1c (HbA1c) were identified as significant factors contributing to cognitive impairment at 7 days and 6 months after CABG in patients with DM (5). In a separate study of 1,010 patients, elevated preoperative HbA1c levels were identified as a risk factor for postcardiac surgery delirium, irrespective of a DM diagnosis, in patients undergoing cardiac surgery (17).

Lachmann et al. (23), in their 2018 observational analysis of three clinical trials involving 1,034 patients, revealed that DM is associated with a 1.84-fold increased risk of POCD. However, obesity, BMI, hypertension, and baseline blood pressure were not found to be associated with POCD in fully adjusted models (23). An observational study identified high glycemic variability as increasing the risk of postoperative delirium (POD) by 38.9% in patients undergoing acute aortic dissection (24). Two studies (25, 33) indicated that preoperative DM is associated with an increased risk of POD (16.83%, 16.7%) in older patients, with low intraoperative alpha power partially mediating this association (33). Seven studies (6, 27, 28, 30, 34, 37, 38) investigated the association of DM with PND, POD, or POCD in non-cardiac surgery, finding that DM patients appear to be at a higher risk of PND after surgery, these pooled observational data consistently showed an association between pre-existing DM and a higher incidence of POD/POCD; causal inference awaits interventional trials (27).

Hyperglycemia was consistently associated with POD regardless of the presence of DM (28). Windmann et al. (37) found that intraoperative hyperglycemia was independently associated with POD but not POCD. Chronic metformin use was associated with slightly and non-significantly less delirium in DM patients (30), preoperative Montreal Cognitive Assessment (MoCA) is an independent risk factor for PND in DM, and the changes in glial fibrillary acidic protein (GFAP) and phosphorylated Tau (p-Tau) in DM patients who experienced PND were significantly higher than in non-DM patients (6). The group with a triglyceride-glucose (TyG) index over 8.678 exhibited a higher incidence of POD (34). Yang et al. (38) demonstrated a significant correlation between low alpha wave power of EEG during operation and the occurrence of POD in elderly patients with DM. Nötzold et al. (26) revealed that cognitive outcomes in the early postoperative period are worse in DM patients, and Panidapu et al. (29) found that dexmedetomidine infusion during the intraoperative period was very effective for perioperative glycemic control and reduction of the incidence of POD (6%) in DM. A study disclosed that tight intraoperative glucose control contributed to a statistically and clinically significant increase in POD (31). Sacli and Kara (32) used advanced neuromonitoring methods that can significantly prevent the decrease in cognitive functions in DM. Van Zuylen et al. (35) found that older adult patients with DM undergoing surgery have an increased risk of delayed neurocognitive recovery (DNR) compared to older adult non-DM patients, but no increased risk of POCD (35). A cohort study from the MIMIC-IV database found that focusing on levels of mean blood glucose (MBG), mean absolute glucose (MAG), glycemic lability index (GLI), and MBG trajectory may be more beneficial to assess the potential risk of POD than the blood glucose level alone (36). A retrospective longitudinal study demonstrated that perioperative hyperglycemia was associated with a new onset of PND (31.7%) (39).

## Discussion

The present investigation offers a comprehensive and up-to-date synthesis of the existing literature concerning the relationship between diabetes mellitus (DM) and postoperative neurocognitive disorders (PND), a topic of paramount importance in the realm of global public health. This review highlights the escalating prevalence of DM and its intricate link with PND, which encompasses postoperative delirium (POD) and postoperative cognitive dysfunction (POCD). The heightened incidence of PND is a matter of grave concern as it profoundly affects postoperative recovery processes, diminishes the quality of life for patients, and inflicts a considerable economic strain on both societal and familial levels.

The review’s bibliometric analysis delves into the global distribution of research efforts on DM in the context of PND, revealing that China, the United States, and Germany are at the forefront of this field. This analysis indicates a concentrated research focus within these specific regions, suggesting that these countries are prioritizing the study of DM’s impact on PND, likely due to the significant implications for patient outcomes and healthcare economics. The prominence of these nations in the research landscape underscores the need for continued international collaboration and knowledge exchange to address this pressing public health challenge.

The extensive and robust network of cooperation among various countries, along with the recurrent use of specific keywords, indicates a widespread and profound global interest in deciphering the intricate relationship between diabetes mellitus (DM) and postoperative neurocognitive disorders (PND). This international collaboration is not only significant but also essential for deepening our comprehension of the multifaceted mechanisms that underlie PND in the context of diabetes.

A systematic analysis of 23 studies, encompassing a substantial cohort of 84,038 patients from diverse geographical regions, underscores the pervasive nature of this issue, as referenced in the literature (5, 6, 17, 20–39). The findings from these extensive studies are strikingly consistent, demonstrating that DM is correlated with an elevated risk of PND, with a particular emphasis on postoperative delirium (POD). For example, Kadoi et al. (5) identified preoperative depression as a notable risk factor for both short-term and long-term postoperative cognitive dysfunction (POCD) in diabetic patients undergoing coronary artery bypass grafting (CABG). This association is further corroborated by Lachmann et al. (23), who reported a 1.84-fold increase in the risk of POCD among DM patients. These findings are particularly concerning, as they suggest that DM impacts not only metabolic processes but also have significant implications for cognitive health during the perioperative period.

The review also delves into the potential mechanisms that link DM and PND, including vascular brain injury (21, 22), impaired brain glucose metabolism (22), inflammation, oxidative stress, and increased levels of tau biomarkers (6). These mechanisms provide a scientific basis for understanding how DM might contribute to PND and suggest potential targets for intervention. For instance, Zhang et al. (19) revealed that the enhancement of lipopolysaccharide (LPS)-induced microglial activation and inflammatory cytokine levels through the toll-like receptor 4 (TLR4)/Janus kinase 2 (JAK2)/signal transducer and activator of transcription 3 (STAT3) pathway offer a novel perspective on the pathophysiological relationship between DM and POCD. Additionally, a preclinical study indicated that mammalian target of rapamycin (mTOR) hyperactivation regulates autophagy, playing a pivotal role in the mechanism underlying PND, and suggests that modulating mTOR signaling could be a promising therapeutic strategy for PND in diabetic patients (8).

Jiao et al. (9) suggest that adult mice with type 2 DM are at an increased risk of developing POCD, potentially due to the downregulation of glutamate transporter-1 (GLT-1) in hippocampal astrocytes. This downregulation enhances the increased glutamatergic neuron excitability induced by anesthesia/surgery, leading to oxidative stress reactions and neuronal apoptosis (9). A study also found that isoflurane anesthesia exacerbates cognitive impairment induced by a single injection of streptozotocin (STZ), likely related to the activation of oxidative stress and inflammatory responses in the rat hippocampus (40). However, Li et al. (10) discovered that insulin treatment restored insulin signaling disrupted by anesthesia by activating the phosphatidylinositol 3-kinase (PI3K)/3-phosphoinositide dependent protein kinase-1 (PDK1)/protein kinase B (AKT) pathway and attenuated anesthesia-induced hyperphosphorylation of tau at multiple Alzheimer’s disease-associated sites. Consequently, intranasal insulin administration might emerge as a promising therapy to prevent anesthesia-induced cognitive deficits in elderly individuals.

Although the review has yielded substantial insights, it is not without its constraints. The studies encompassed within the review exhibit considerable heterogeneity, which is evident in their diverse methodologies, participant demographics, and the surgical interventions they describe. This variability could potentially undermine the broader applicability of the findings. Moreover, the preponderance of the research has concentrated on postoperative delirium (POD), with a comparatively smaller subset examining postoperative cognitive dysfunction (POCD). This imbalance may lead to a biased perspective on the comprehensive spectrum of postoperative neurocognitive disorders (PND) in patients with diabetes mellitus (DM).

Moreover, the review’s scope did not encompass critical variables such as the duration of DM, the specific type of DM, or the precise anti-diabetic therapeutic regimens, all of which could significantly influence the risk profile for PND. These factors, when unaccounted for, may obscure the true relationship between DM and PND. To mitigate these limitations, future investigative efforts should endeavor to conduct expansive, multicenter, and longitudinal studies. Such research endeavors would be better positioned to clarify the causal nexus between DM and PND.

It is also imperative that forthcoming studies delve into the distinct impacts of type 1 versus type 2 DM on PND. This includes scrutinizing the diverse effects of various anti-diabetic medications and assessing how stringent glycemic control might influence the onset of PND. Additionally, interventional research is sorely needed to assess the effectiveness of strategies designed to optimize glycemic control in the perioperative period and to potentially decrease the incidence of PND among diabetic individuals. These studies should aim to provide actionable insights that could inform clinical practice and improve patient outcomes. Until randomized trials are available, clinicians should: (a) include DM status and pre-operative HbA1c in delirium-risk stratification, (b) monitor glycemic variability—not just absolute glucose—throughout the peri-operative period, and (c) involve geriatrics or psychiatry early when DM patients screen positive for pre-operative depression or cognitive impairment.

## Conclusion

To sum up, this analysis emphasizes the substantial correlation between diabetes mellitus (DM) and perioperative neurocognitive disorders (PND), underscoring the imperative for additional investigative efforts and clinical focus. Given the escalating global incidence of DM, comprehending and tackling the nexus between DM and PND is becoming more crucial for the enhancement of patient outcomes and the mitigation of the healthcare system’s strain. Because the included studies are observational and geographically clustered, causality cannot be inferred and findings may not generalize to populations with differing peri-operative care pathways; large, multi-center trials that control diabetes type, duration and medication strata are still required.

## Data Availability

The original contributions presented in this study are included in this article/Supplementary material, further inquiries can be directed to the corresponding author.
