# Supplementary material for: Diabetes mellitus is associated with an increased risk of postoperative neurocognitive disorders: a systematic review
Source: Front Med (Lausanne). 2026 Feb 13;13:1726908. doi: 10.3389/fmed.2026.1726908 (PMC12946032; doi:10.3389/fmed.2026.1726908)
Supplement: Supplementary file 1 [file Data_Sheet_1.docx]

Pubmed 11/15/24: 46

| **#1 (((postoperative delirium [Title/Abstract]) OR (postoperative cognitive decline [Title/Abstract])) OR (postoperative cognitive dysfunction [Title/Abstract])) OR (postoperative neurocognitive disorders [Title/Abstract])** |
| --- |
| **#2(Preoperative diabetes [Title/Abstract]) OR ("Diabetes Mellitus"[Mesh])** |
| #3 #1 AND #2 |

Web of science 11/15/24: 182

| #1((((TS=(Postoperative neurocognitive disorders)) OR TS=( Perioperative neurocognitive disorders)) OR TS=(Postoperative delirium)) OR TS=(Postoperative cognitive decline)) OR TS=(Postoperative cognitive dysfunction) |
| --- |
| #2((TS=(Preoperative diabetes)) OR TS=(Diabetes)) OR TS=(Diabetes mellitus) |
| #3 #1 AND #2 |

Embase 11/15/24: 492

| #1**'postoperative delirium' or 'postoperative cognitive dysfunction' or 'postoperative cognitive decline' or 'postoperative neurocognitive disorders'** |
| --- |
| #2**'diabetes mellitus' or 'preoperative diabetes'** |
| #3 #1 AND #2 |

Cochrane 11/15/24: 1820

| #1(postoperative delirium):ti,ab,kw or (postoperative cognitive decline):ti,ab,kw or (postoperative cognitive dysfunction):ti,ab,kw or (Preoperative diabetes):ti,ab,kw |
| --- |
| #2(Diabetes mellitus):ti,ab,kw or (Preoperative diabetes):ti,ab,kw |
| #3 #1 AND #2 |
